# **Corticosteroid enhances epithelial barrier function in intestinal organoids derived from patients with Crohn’s disease**

**DOI:** 10.1007/s00109-021-02045-7

**Published:** 2021-02-11

**Authors:** Pan Xu, Montserrat Elizalde, Ad Masclee, Marieke Pierik, Daisy Jonkers

**Affiliations:** 1 Division of Gastroenterology-Hepatology, Department of Internal Medicine, Maastricht University Medical Centre+, P. Debyelaan 25 6229 HX, Maastricht, the Netherlands; 2 School of Nutrition and Translational Research in Metabolism, Maastricht University, Maastricht, the Netherlands

**Keywords:** Corticosteroids, Intestinal organoids, Barrier function

## Abstract

**Abstract:**

Corticosteroids (CS), first-line therapeutics for Crohn’s disease (CD) with moderate or severe disease activity, were found to restore intestinal permeability in CD patients, whereas the underlying molecular events are still largely unknown. This study aimed to investigate the effect and mechanisms of CS prednisolone on epithelial barrier using CD patient-derived intestinal organoids. 3D intestinal organoids were generated from colon biopsies of inactive CD patients. To mimic the inflammatory microenvironment, a mixture of cytokines containing TNF-α, IFN-γ, and IL-1β were added to the organoid culture with or without pre-incubation of prednisolone or mifepristone. Epithelial permeability of the organoids was assessed by FITC-D4 flux from the basal to luminal compartment using confocal microscopy. Expression of junctional components were analyzed by qRT-PCR, immunofluorescence staining, and western blot. Activity of signaling pathways were analyzed using western blot. Exposure of the cytokines significantly disrupted epithelial barrier of the intestinal organoids, which was partially restored by prednisolone. On the molecular level, the cytokine mixture resulted in a significant reduction in E-cadherin and ILDR-1, an increase in CLDN-2, MLCK, and STAT1 phosphorylation, whereas prednisolone ameliorated the abovementioned effects induced by the cytokine mixture. This study demonstrates that prednisolone confers a direct effect in tightening the epithelial barrier, identifies novel junctional targets regulated by prednisolone, and underscores intestinal barrier restoration as a potential mechanism that contributes to the clinical efficacy of prednisolone in CD patients.

**Key messages:**

- Prednisolone confers a direct preventive effect against cytokine-induced barrier dysfunction.
- Prednisolone regulates the expression of CLDN-2, E-cadherin, and ILDR-1.
- The effect of prednisolone is GR-, MLCK-, and STAT1-dependent.

**Supplementary Information:**

The online version contains supplementary material available at 10.1007/s00109-021-02045-7.

## Introduction

The intestinal epithelium functions as a permeable and dynamic interface that permits the absorption of water, electrolytes, and dietary nutrients, while also serving as a critical barrier that protects the intestinal surface from numerous microorganisms and foreign antigens that harbor in the gut lumen [1, 2]. The integrity of gut barrier is maintained through the formation of complex protein-protein networks that mechanically link neighboring cells and regulate paracellular permeability. On the ultrastructural level, these are composed of three types of junctional complexes: tight junctions (TJs), adherens junctions (AJs), and desmosomes [3]. Over the last decade, a strong correlation has been established between disrupted gut barrier and the presence of several inflammatory disorders, such as Crohn’s disease (CD). CD belongs to the inflammatory bowel diseases (IBD), and is characterized by chronic recurrent inflammation of the gastrointestinal tract [4]. A defective intestinal barrier has emerged as an important pathogenic factor contributing to the development and progression of CD. It is proposed that a disrupted mucosal barrier results in increased intestinal permeation of luminal toxins and triggers an immunological response that promotes intestinal inflammation. As part of enhanced inflammation, systemic concentrations of several cytokines including e.g. TNF-α, IFN-γ, and IL-1β, were found to be markedly increased in patients with CD when compared to healthy control subjects [5], and were shown to disrupt epithelial barrier in different cells or animal models. In fact, a comprised intestinal permeability has been well recognized in CD patients and correlates with disease activity [6–9]. Altered expression of different TJs and AJs were also noted in patients with CD [10]. In particular, a few studies demonstrated that medical therapies that tighten a disrupted epithelial barrier were shown to promote the resolution of active inflammation in CD patients [11, 12], demonstrating that strengthening a leaky gut barrier is a potential therapeutic strategy in CD therapy.

Systemic corticosteroid (CS), i.e. prednisolone, is indicated as first-line therapeutic for the treatment of CD patients with moderate to severe disease activity. CS is efficient in improving the inflammatory responses through a variety of mechanisms, including suppression of cytokine gene transcription, eicosanoid biosynthesis, and intercellular adhesion molecules [13]. Moreover, one study showed that CS therapy restores disrupted intestinal barrier function in CD patients [14]. However, the molecular events underlying the CS actions are still not fully understood. In particular, it remains largely unclear whether CS, in addition to its anti-inflammatory effects on reducing inflammatory mediators that regulate barrier function, confers a direct effect on tightening the epithelial barrier in the human intestinal epithelium.

Therefore, the main aim of this study is to investigate the effect of corticosteroid prednisolone on intestinal barrier and to elucidate the underlying molecular mechanisms using CD patient-derived intestinal organoid model. Our results demonstrate that prednisolone confers a direct preventive effect against cytokine-induced barrier dysfunction. This beneficial effect is glucocorticoid receptor (GR)-dependent, and is accompanied by normalized expression of CLDN-2, ILDR-1, and E-cadherin on both mRNA and protein levels, through regulating the MLCK and STAT1 signaling pathways. Collectively, we provided insights into the mode of action of prednisolone in tightening epithelial barrier function, and the mechanism that contributes to the therapeutic efficacy of corticosteroids in the treatment of CD.

## Methods and materials

### Patient inclusion

The inclusion of CD patients is based on the IBD South Limburg (IBDSL) cohort study, which has been approved by the Medical Ethics Committee of the Maastricht University Medical Center+ (MUMC+), and was conducted in accordance with the Declaration of Helsinki (Seoul, South Korea, Oct. 2008). All subjects signed informed consent before participation. Intestinal biopsies were collected from the uninflamed ascending colon in patients undergoing diagnostic endoscopy with CD involved in the terminal ileum (L1 according to the Montreal classification), colon (L2), or ileocolon (L3). Characteristics of the participants are listed in Supplementary Table 1.

### Human intestinal crypt isolation and organoid culture

Intestinal organoids were established and cultured following previously described protocol with minor modifications [15]. In short, directly after collection, the biopsies were washed four times with cold 1% antibiotic-antimycotic (Invitrogen, CA, USA) in PBS for 2 min, followed by three times of washings with 10 mM DTT/PBS for 2 min. Thereafter, the samples were incubated with 2 mM EDTA in PBS for 1 h (4 °C, 5 rpm). After removal of EDTA, the biopsies were vigorously shaken several times in cold PBS to obtain a supernatant fraction that contains intestinal crypts. To collect RNA from the crypts, small amount of supernatant that contains around 100 crypt particles was portioned, and the remaining supernatant was used for organoid generation. The supernatants were centrifuged at 400*g* for 8 min at 4 °C to enrich crypt particles. To generate organoids, the crypt pellets were washed with cold basal medium (DMEM/F12 medium containing 1% GlutMax, 1% Hepes, and 5% FBS), centrifuged at 400*g* for 3 min at 4 °C, and then embedded in Matrigel hESC-Qualified LDEV-free Matrix (Corning BV, Amsterdam, the Netherlands) on a pre-warmed 4-well glass chamber (Ibidi GmbH, DE) at 37 °C, and then supplemented with IntestiCult™ Organoid Growth Medium (Stem Cell Technology, GmbH, DE) to form intestinal organoids. Organoids were maintained in a 37 °C 5% CO_2_ atmosphere with media changed every 3 days. After around 15 days of culture, primary intestinal organoids exhibit a mature morphology with thickened epithelium layer, multiple lumens, and columnar buddings. Those organoids were then passaged following previously described procedures [16]. At around day 5 to 7, the organoids grow into hollow morphology with a single layer of cells, which allow real-time barrier function assessment [17].

### Cytokines and chemicals exposed to intestinal organoids

Recombinant human cytokines TNF-α, IFN-γ, and IL-1β were purchased from Sigma-Aldrich (Saint Louis, USA). Prednisolone was obtained from PeproTech (Rocky Hill, USA). Clarithromycin (CAM) was kindly provided by the Hospital Pharmacy of MUMC+. Mifepristone (RU486) was purchased from Sigma-Aldrich. Intestinal organoids were exposed basolaterally to a cytokine complex containing TNF-α, IFN-γ, and IL-1β (20 ng/mL of each) for 24 h, with or without the incubation of prednisolone (10 μM), clarithromycin (80 μM), or mifepristone (10 μM) for 12 h at 37 °C. Control wells were left untreated.

### Paracellular permeability measurement in intestinal organoid

Upon the indicated exposure, paracellular permeability was determined by co-incubating organoids with 1 mg/mL, 10% (v/v) FITC-D4 (Sigma) at 37 °C for 24 h. Permeation of the marker FITC-D4 from the basal to luminal side of the organoids was assessed using confocal microscopy (Leica Microsystems GmbH, DE) and the Image J software. Permeability was determined by calculating the luminal/basolateral ratio (L/BL) based on the density of FITC-D4 in the lumen and basal compartment. Given the high biological variance of the organoids within each group as reflected by the variable size of individual organoid, at least 25 organoids (with diameter within 100 ~ 150 μm) were included per group for quantification.

### RNA extraction and quantitative RT-PCR analysis

Total RNA was isolated from intestinal crypts and organoids using RNeasy Micro Kit (Qiagen, Valencia, CA) or from Caco-2 monolayers using the RNeasy Mini Kit (Qiagen) according to the manufacture’s protocol. For reverse transcription, 500 ng RNA from intestinal crypts and organoids or 1 μg RNA from Caco-2 cells was used to convert into cDNA using iScript Select cDNA Synthesis Kit (Bio-Rad, CA, USA). Quantitative PCR was performed via CFX384 Real-Time PCR Detection System (Bio-Rad) using SYBR Green Supermix (Bio-Rad) according to the manufacturer’s instructions. For GR expression analysis, pre-designed TaqMan gene expression assays (Applied Biosystems, Amsterdam, the Netherlands) targeting GR-α (cat. # Hs00230818_m1), GR-β (cat. # Hs00354508_m1), and housekeeping gene HPRT (cat. # Hs02800695_m1) were used following the manufacturer’s instructions. Primer sets used are listed in Supplementary Table 2. The relative expression of the target genes was normalized to the housekeeping gene 18S.

### Protein extraction and western blot

After the exposure, intestinal organoids were lysed in 100 μL RIPA buffer and the extracted proteins were denatured using SDS loading buffer (125 mM Tris–HCl pH 6.8, 4% sodium dodecyl sulfate, 20% glycerol, 0.04% bromophenol blue, and 100 mM β-mercaptoethanol) at 95 °C for 5 min. For each sample, 10 μg protein was loaded and separated on 10% or 12% mini-protein TGX precast protein gels (Bio-Rad) and transferred to polyvinylidene difluoride membranes (GE Healthcare, Chicago, USA). The membranes were incubated overnight at 4 °C with appropriate primary antibodies, subsequently with specific secondary antibodies and visualized using the enhanced chemiluminescence reagent (Thermo Scientific). The following antibodies were used: Rabbit anti-alpha-TUBULIN, mouse anti-E-cadherin, and rabbit anti-NF-κB p65 were from Abcam; rabbit antibodies against p38, phospho-p38, JNK, phospho-JNK, ERK, phospho-ERK, MLC, phosphor-MLC, phospho-NF-κB p65, STAT1, and phospho-STAT1 (Tyr701) were from Cell Signalling Technology; mouse anti-CLDN-2 and rabbit anti-ILDR-1 were from Invitrogen; anti-mouse/rabbit IgG horseradish peroxidase-linked secondary antibodies were from Cell Signalling Technology. Original western blot images were included in Supplementary Figures 7, 8, and 9. Densitometric quantification analyses of the western blots were performed using the Image J software.

### Immunofluorescence staining and imaging of junctional proteins

After the exposure, organoids were rinsed with PBS, fixed with 4% (w/v) paraformaldehyde, permeabilized with 0.5% (v/v) Triton X-100 in PBS at RT for 30 min, and then blocked with 1% (w/v) BSA at RT for 1 h. Afterwards, the organoids were incubated at 4 °C for 36 h with mouse anti-CLDN-2 (1:100 dilution, Invitrogen) or overnight with mouse anti-E-cadherin (1:200 dilution, Abcam) following 1-h incubation of Alexa Fluor 488 goat anti-mouse IgG secondary antibody (1:200 dilution, Thermo Fisher Scientific). Organoids were then washed three times with PBS and stained with diamidino-2-phenylindole (DAPI, Sigma) at 1:1500 dilution. After another two washings with PBS, organoids were mounted using VectaShield mounting medium (Vector Laboratories, Burlingame, USA). Confocal images were obtained using a confocal microscopy (Leica Microsystems GmbH) with identical acquisition settings (laser power, objectives, magnifications) for each acquired image and condition. Images were then analyzed using the Image J software.

### Cell death assay

Cell death assay was determined by measuring the release of lactate dehydrogenase (LDH) into the culture medium using the LDH assay kit (CytoTox ONEtm; Promega, the Netherlands) according to manufacturer’s instructions. Maximum LDH release was induced by using lysis solution. The percentage of LDH activity was calculated as the percentage of the maximum LDH release from fully lysed cells.

### Caco-2 cell culture

Human epithelial Caco-2 cells were obtained from ATCC (Rockville, USA) and were cultured (passage 47 to 57) in Dulbecco’s modified Eagle’s medium (DMEM, Lonza Benelux BV, NL), with 10% (v/v) fetal calf serum (FBS, Invitrogen), 1% (v/v) solution of non-essential amino acids (NEAA, Invitrogen), and 1% (v/v) solution of antibiotic-antimycotic mixture (anti-anti, Invitrogen) in an atmosphere of 5% CO_2_ at 37 °C.

### Statistical analysis

Data are expressed as mean values ± standard error of the mean (SEM) and indicating *n* as the number of biological samples or measurements. All FITC-D4 permeability assays, qRT-PCR, and western blots were performed with at least three independent biological replicates and three technical replicates for each reaction. Statistical analysis was performed using Student’s *t*-test or one-way ANOVA and Tukey’s post hoc test (Prism 6 GraphPad). Significant differences between two groups were noted by asterisks (*p < 0.05, ** *p* < 0.01, ***p < 0.001).

## Results

### Prednisolone ameliorates intestinal barrier disrupted by pro-inflammatory cytokines

**Fig. 1:**
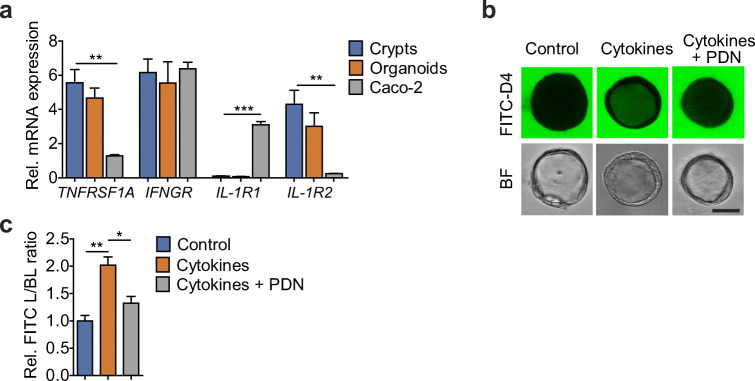
Prednisolone ameliorates intestinal barrier disrupted by pro-inflammatory cytokines. **a** Expression levels of TNFRSF1A, IFNGR, IL-1R1, and IL-1R2 receptors, calculated as percentage (%) relative to 18S, in colonic crypts (*n* = 4) and organoids (*n* = 4) and Caco-2 cells (*n* = 4) analyzed by RT-PCR. Data expressed as means ± SEM. **p < 0.01 and ***p < 0.001 by Student’s *t*-test. **b**-**c** Representative FITC-D4 permeation or bright-field (BF) microscopy images (**b**) and quantification of FITC-D4 permeation (**c**) in control (*n* = 6) and cytokine cocktail (20 ng/mL each of TNF-α, IFN-γ, and IL-1β, *n* = 6) treated intestinal organoids. The bar indicates 50 μm. The mean fluorescence intensity of FITC-D4 was measured and expressed as the L/BL ratio of the luminal (L) over the basal (BL) compartment. Data expressed as means ± SEM with at least 25 organoids per subject and 6 subjects per group. *p < 0.05 and **p < 0.01 by one-way ANOVA and Tukey’s post hoc test

We previously observed that intestinal organoids from active and inactive CD patients showed no difference in baseline epithelial permeability (unpublished data). This is in line with previous report indicating the loss of inflammatory status from the intestinal tissue to the stem cell-derived organoids [18]. To recreate a more physiological microenvironment, we used a cytokine cocktail that contains TNF-α, IFN-γ, and IL-1β (20 ng/mL each), the aberrant productions of which have been implicated as critical contributors in perpetuating intestinal inflammation in CD [19]. First, the expression levels of receptors for TNF-α, IFN-γ, and IL-1β in CD patient-derived intestinal organoids were evaluated. QRT-PCR analysis demonstrated that TNFRSF1A and IFNGR and IL-1R1/2 showed similar mRNA expression in the organoids as compared to the colonic crypts, while most of those receptors showed significantly different profiles in Caco-2 cells (Fig. 1a). To further investigate the effect of these pathophysiological stimuli on epithelial barrier, intestinal organoids were exposed to the cytokine cocktail for 24 h. FITC-D4 was also added in the culture medium for real-time barrier function evaluation. Under confocal microscopy, we observed an increased intraluminal FITC-D4 signal post cytokine exposure compared to the control treatment (Fig, 1b-c, 1.000 ± 0.098 vs. 2.021 ± 0.151, *p* < 0.05).

In one previous study, CS was shown to restore an increased intestinal permeability as determined by the urinary lactulose/mannitol ratio in CD patients [14]. To verify whether a similar effect could be observed in the in vitro organoid culture, synthetic prednisolone was chosen as an example and exposed to the organoids at 10 μM post cytokine cocktail treatment. Under unchallenged condition, prednisolone exposure did not alter basal FITC-D4 permeation of the organoids (Supplementary Figure 1), but significantly reduced intraluminal FITC-D4 signals that were increased by cytokine cocktail (Fig. 1b-c, 2.021 ± 0.1505 vs. 1.321 ± 0.1271, *p* < 0.05). In contrast, clarithromycin (CAM), an antibiotic that can be used to treat primary active CD, exhibited no effect in ameliorating the disrupted epithelial barrier (Supplementary Figure 2), indicating that the epithelial barrier of intestinal organoids is functionally dynamic to stimuli and selectively responds to different therapeutics.

**Fig. 2:**
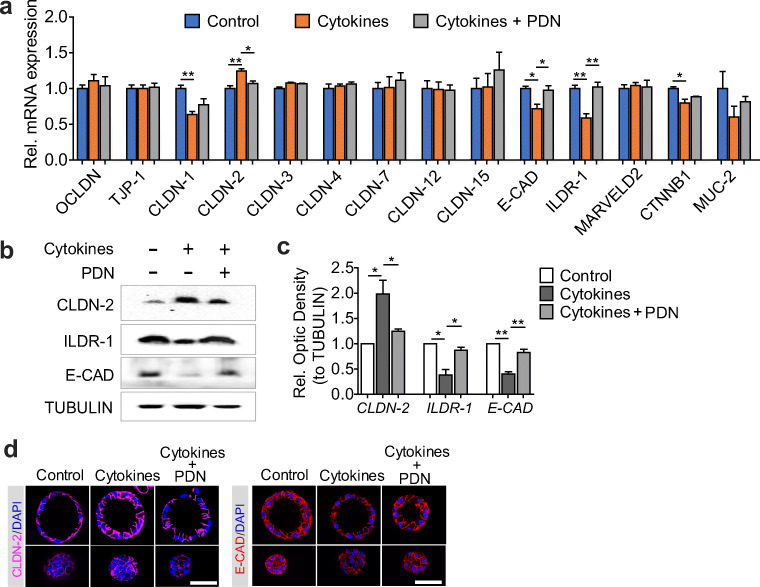
Effects of prednisolone on epithelial junctional components. Intestinal organoids were exposed basolaterally with or without cytokine cocktail (20 ng/mL TNF-α, IFN-γ, and IL-1β, *n* = 6) for 24 h, with or without prednisolone incubation (10 μM, *n* = 6) for 12 h. **a** Transcriptional expression of various junctional genes in the intestinal organoids. **b**-**d** Representative western blots (**b**), optical densitometric quantification (**c**), and immunofluorescence images (**d**) of CLDN-2, ILDR-1, and E-cadherin (E-CAD) in the organoids. Images in **d** were captured from the middle cross section (upper panel) or top section (lower panel) of the organoid. Bar indicates 50 μm. Data expressed as means ± SEM. *p < 0.05 and **p < 0.01 by one-way ANOVA and Tukey’s post hoc test

To evaluate whether altered FITC-D4 permeation was due to cell death, we first performed lactate dehydrogenase (LDH) assay to evaluate the effects of cytokine cocktail or prednisolone on cell viability. Triton X-100 was used as a positive control to induce maximum LDH leakage. Compared to the control treatment, no significant difference in LDH activity was detected post cytokine cocktail or prednisolone treatments (Supplementary Figure 3A). In addition, qRT-PCR analysis revealed no significant changes on the expression of apoptosis markers BAX and BCL-2, as well as necroptosis markers MLKL and IL-8 in the intestinal organoids by cytokine cocktail or prednisolone stimulations (Supplementary Figure 3B).

### Prednisolone decreases CLDN-2 and increases E-cadherin, ILDR-1

To further investigate the molecular effects of prednisolone, we performed qRT-PCR analysis to evaluate the expression of receptors TNFRSF1A and IFNGR and IL-1R1/2, which showed no significant changes upon prednisolone exposure compared to the control treatment (Supplementary Figure 4). We then evaluated whether prednisolone exerts a direct effect on the epithelial barrier junctional components. RT-PCR analysis showed that 24-h treatment of the cytokine cocktail resulted in a significant decrease of claudin (CLDN)-1, E-cadherin (E-CAD), ILDR-1, and CTNNB1, accompanied by an increase in CLDN-2 on the mRNA levels (Fig. 2a). The transcripts of OCCLUDIN (OCLDN), TJP-1, CLDN-3, CLDN-4, CLDN-7, CLDN-12, CLDN-15, MUC-2, and MARVELD2 were not affected by the stimulation of pro-inflammatory cytokines (Fig. 2a). Incubation of prednisolone (10 μM) could partially restore the altered expression of CLDN-2, E-CAD, and ILDR-1 (Fig. 2a). These findings were also confirmed on protein level as evidenced by western blot analysis and immunofluorescence staining (Fig. 2b-d). In addition, no significant distortion of CLDN-2 and E-CAD belts was observed (Fig. 2d).

### Prednisolone augments epithelial barrier function in a GR-dependent manner

**Fig. 3:**
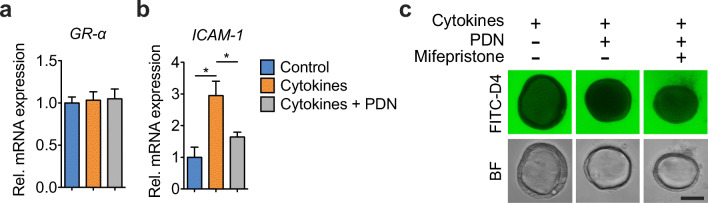
The effect of prednisolone on epithelial barrier function is GR-dependent. Intestinal organoids were exposed basolaterally with or without the cytokine mixture (20 ng/mL TNF-α, IFN-γ, and IL-1β, *n* = 6) for 24 h, with or without 10 μM prednisolone pre-incubation for 12 h. **a**-**b** Transcriptional expression of GR-α and ICAM-1 genes in the intestinal organoids. Data expressed as means ± SEM. *p < 0.05 by Student’s *t*-test. **c** Representative FITC-D4 permeation or bright-field (BF) microscopy images of the intestinal organoids upon the abovementioned treatments. Bar indicates 50 μm

We then examined whether the beneficial effect of prednisolone on epithelial barrier was mediated via the intracellular activation of glucocorticoid receptor (GR) on its expression or activity levels. QRT-PCR analyses demonstrated that GR-β showed undetectable expressing level in intestinal organoids. GR-α isoform was abundantly expressed but not significantly changed under the cytokine cocktail or prednisolone exposures (Fig. 3a). To evaluate the activity of GR signaling, we analyzed the transcript of ICAM-1, which is demonstrated to be expressed in human epithelial cells [20] and serves as a downstream target marker for GC responsiveness [21, 22]. We observed that cytokine cocktail exposure resulted in a significant increase of the ICAM-1 transcripts, while prednisolone treatment hampered this induction (Fig. 3b). In addition, administration of 10 μM mifepristone, a molecule that specifically inhibits GR transactivation, repressed the beneficial effects of prednisolone in restoring the cytokine-induced epithelial barrier disruption (Fig. 3c and Supplementary Figure 5) indicating that the effect of prednisolone on epithelial barrier function is GR-dependent.

### Prednisolone treatment decreases the activity of MLCK and STAT1 signalings

**Fig. 4:**
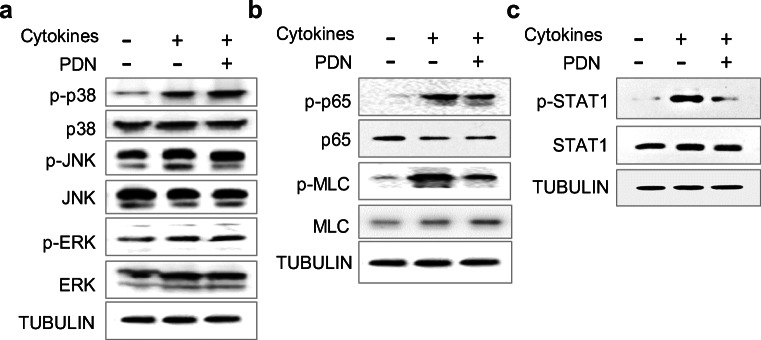
Prednisolone decreases the activity of MLCK and STAT1 signaling pathways. **a**-**c** Protein levels of phospho (p)-p38, p38, p-JNK, JNK, p-ERK, ERK (**a**), p-p65, p65, p-MLC, MLC (**b**), p-STAT1, and STAT1 (**c**) in intestinal organoid with or without the treatment of cytokine mixture (20 ng/mL TNF-α, IFN-γ, and IL-1β) for 24 h, with or without 10 μM prednisolone incubation for 12 h

We then performed western blot to analyze the involvement of MAPK, MLCK, and STAT1 signaling pathways, which were shown to be downstream cascades responding to GR transactivation in IBD patients, and have been implicated in the regulation of epithelial barrier function in immobilized cells. As shown in Fig. 4a and Supplementary Figure 6A, cytokine exposure increased the phosphorylation levels of MAPK p38, but not p-JNK and p-ERK. However, treatment of prednisolone showed no significant effect on p38 phosphorylation. Notably, we observed a significant activation of MLCK downstream targets MLC and NF-kB p65 isoform, and an increased phosphorylation of STAT1 when the organoids were exposed to the cytokine mixture. The increase in p-MLC, p-p65, and p-STAT1 was further prevented by prednisolone (Fig. 4b-c, Supplementary Figure 6B-C), which suggested a distinct involvement of the MLCK and STAT1 signalings in the mediation of epithelial barrier function by prednisolone.

## Discussion

**Fig. 5:**
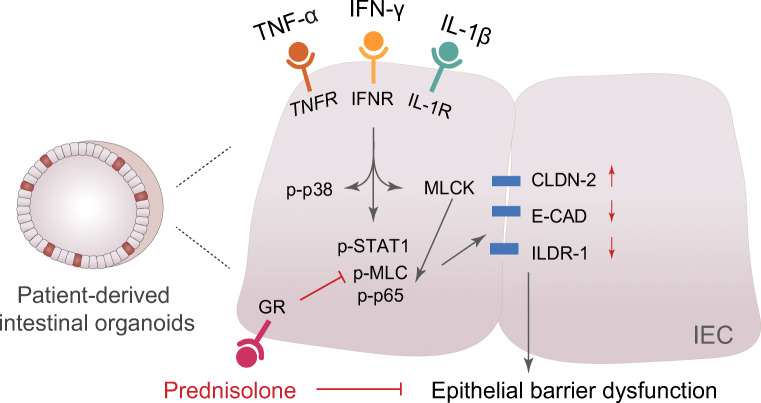
Graphical summary. Prednisolone restores epithelial barrier function attenuated by combined cytokines TNF-α, IFN-γ, and IL-1β in CD patient-derived intestinal organoids. On the molecular level, this effect is GR-dependent, and involves MLCK and STAT1 signaling pathways, through a regulation of junctional components CLDN-2, E-CAD (E-cadherin), and ILDR-1

In the present study, using a CD patient-derived 3D intestinal organoid model, we evaluated the effects and underlying mechanisms of prednisolone on epithelial barrier function. In the organoids, the expression of cytokine receptors TNFRSF1A and IFNGR and IL-1R1/2 were detected and showed similar mRNA levels as that in the colonic crypts. Exposure of a cytokine mixture containing 20 ng/mL each of TNF-α, IFN-γ, and IL-1β triggered an increased in paracellular permeability, accompanied by a disruption of epithelial junctional components. These effects could be partially rescued by prednisolone in a GR-dependent manner, through modulating CLDN-2, E-cadherin, and ILDR-1 expression, with the involvement of MLCK and STAT1 signaling pathways (Fig. 5).

Previous studies showed that CD patients often have elevated levels of various pro-inflammatory cytokines, including e.g. TNF-α, IFN-γ, and IL-1β, and increased intestinal permeability [5–9]. In immortalized cells, TNF-α, IFN-γ, and IL-1β are able to disrupt epithelial barrier function, while the mechanistic regulations of those cytokines on junctional components vary (often conflict) between different studies, which could be associated with the cell models that were applied. For instance, TNF-α induces CLDN-1 expression in IEC-18 cells [23], but reduces CLDN-1 expression in Caco-2 cells [24]. In T84 cells, TNF-α/IFN-γ reduces CLDN-2 expression [25], while their combination induces CLDN-2 in Caco-2 cells [26]. In T84 monolayer, IFN-γ induces a significant loss of paracellular integrity through regulating CLDN-1 and OCLDN expression, whereas no effect is observed by the same authors in Caco-2 cell monolayer [27]. Notably, most of the previous findings on cytokine-regulated epithelial barrier function were performed using immortalized colorectal cancer cell lines, especially the Caco-2 cells, which are known to have tighter cell junctions and are more resistant to stressors than the human intestinal epithelium [28, 29]. Benefiting from the recently advanced stem cell technologies, ex vivo culture of patient-derived intestinal organoid has been shown to be more physiological compared to the widely used immortalized cells [30]. In comparison to the widely used Caco-2 cells, patient-derived intestinal organoids were shown to develop a multicellular phenotype [31–33]. RNA-seq analysis also revealed that the transcriptional profile of primary intestinal organoids was distinct from Caco-2 cells [34], which is in line with our observation that the receptors for TNF-α, IFN-γ, and IL-1β were differently expressed in intestinal organoids and Caco-2 cells. In addition, a cytokine cocktail was applied in our study, which most likely better reflects the endogenous cellular microenvironment, as the target cells are exposed to a variety of cytokines under physiological inflammatory conditions and synergistic effects were reported upon stimulation of combined cytokines [35–38]. Different from most of the previous studies that exposed the cytokine cocktail to immortalized cell lines, we hereby applied the cocktail in the patient-derived intestinal organoids. As a result, we observed that exposure of the cytokine complex compromises epithelial barrier function in human intestinal organoids, supporting the notion that TNF-α, IFN-γ, and IL-1β, in addition to their well-established function in regulating inflammatory responses, have a crucial pathological effect on intestinal barrier function. The detrimental effects of cytokines on epithelial permeability are in line with previous reports from immortalized colorectal cell lines. However, on the molecular level, we revealed that the cytokine cocktail treatment resulted in a significant decrease of CLDN-1, E-cadherin, CTNNB1, and ILDR-1, accompanied by an increase in CLDN-2. Previous studies showed that CLDN-1 is a downstream target of cytokine TNF-α in Caco-2 cells [24] and IFN-γ in T84 cells [39]. It has also been demonstrated that TNF-α, IFN-γ, or IL-1β regulate CLDN-2 expression in HT-29 cells [40], T84 cells [41], or Caco-2 cells, respectively [26]. To the best of our knowledge, we for the first time identified E-cadherin, CTNNB1, and ILDR-1 as junctional genes that are regulated by the cytokine mixture in the human intestinal epithelium. Notably, we previously have identified OCLDN and TJP-1 as downstream targets regulated by TNF-α in Caco-2 cells [42]. As a contrast, this effect was not observed in patient-derived organoids by TNF-α (unpublished data) or cytokine cocktail.

Previous studies on corticosteroids in CD have mainly revealed their anti-inflammatory or immune-suppressive functions. Although one study showed that treatment with prednisolone resulted in a significant decrease of epithelial permeability as measured by the lactulose/mannitol ratio, little is known on their direct effect on epithelial integrity and underlying mechanisms. In the current investigation, we showed that CD patient-derived intestinal organoid strongly expresses glucocorticoid receptor (mainly the GR-α isoform), the activity of which could be induced by prednisolone. Incubation of prednisolone prevented the barrier disruption induced by the cytokine cocktail, and this effect was abolished by pre-incubation with the GR antagonist mifepristone, indicating that the beneficial effect of prednisolone is GR-dependent. Within the included six inactive CD patients, one received steroid budesonide at the time of biopsy collection, while the other five patients did not receive any steroid treatment in the past 5 years at the time of biopsy collection. In the intestinal organoids derived from those patients, we did not observe significant differences on their GR expression levels, as well as their response to cytokine cocktail or prednisolone. This is most likely due to the fact that the intestinal organoids are developed from multipotent stem cells residing at the base of intestinal crypts. When cultured in the presence of specific conditioned medium, the epithelial stem cell containing crypts will lose the inflammatory characters [18], which are present in the biopsy origin and shown to be related to glucocorticoid responsiveness [13, 43]. While intestinal organoids represent as a novel complementary model to dissect the pathogenic and therapeutic molecular events associated with CD disease, further development of the model is needed to expand its translational potential (i.e., drug prediction).

So far, only a few studies have investigated the effect and molecular regulation of junction complexes in response to glucocorticoid exposure in cultured cell models. For instance, in human retinal endothelial cells, hydrocortisone and dexamethasone induced OCLDN and CLDN-5 expression [44]. Dexamethasone treatment enhanced the epithelial barrier function and promoted ZO-1 distribution along the plasma membrane in human corneal epithelial cell line [45], while it promotes apical tight junction reorganization in rat mammary epithelial tumor cells through mediating the Ras-fascin axis [46, 47]. Data in intestinal epithelial cells are limited. In Caco-2 cells, Fischer et al. showed that dexamethasone attenuates CLDN-2 expression that is upregulated by TNF-α/IFN-γ treatment [26]; Boivin et al. revealed that prednisolone and dexamethasone prevented barrier dysfunction induced by TNF-α [48]. Here, using CD patient-derived intestinal organoids, we showed that prednisolone suppressed CLDN-2 and restored E-cadherin and ILDR-1 expression that was impaired by the cytokine mixture. CLDN-2, a member of the claudin family, regulates paracellular water permeability and ion selectivity by forming cation-selective pores. Expression of CLDN-2 is highly upregulated in colonic biopsies of CD patients [49, 50]. In the present study, the observed effect of prednisolone on CLDN-2 in the intestinal organoids is akin to previous findings by Fischer et al. [26]. In contrast to the finding by Bardenbacher et al., who showed a reduced full-length CLDN-2 protein and an increased CLDN-2 cleavage fragment in murine small intestinal organoids upon IFN-γ treatment [51], we did not observe a cleaved CLDN-2 fragment in human colonic organoids upon cytokine mixture exposure. We speculate this difference could be attributed to the different models or cell lysate fragmentation protocols that were applied in our and their studies. Furthermore, ILDR-1 (encoding gene for angulin-2) belongs to the family of tricellular tight junction, which is a specialized structure that seals the extracellular space between epithelial cells at tricellular contacts (TCs). In cultured epithelial cells, ILDR-1 recruits tricellulin to TCs, where it is required for the establishment of a strong barrier of the epithelium [52]. As a recently discovered tight junction protein, the biological functions of ILDR-1 in epithelial cells are still largely unclarified. Mutations in *ILDR-1* were found to be associated with nonsyndromic autosomal recessive hearing impairment [53]. Another report showed that ILDR-1 contributes to fish gill epithelium barrier properties [54]. Herein, our data revealed that ILDR-1 transcripts were detected in patient-derived intestinal organoids. In particular, we for the first time showed that ILDR-1 is a downstream target of the pro-inflammatory cytokines and prednisolone, unraveling its new regulatory mechanisms in the maintenance of intestinal epithelial barrier function. Further investigations are needed to better understand the involvement of ILDR-1 in IBD pathogenesis. In addition, we also identified E-cadherin as another novel downstream target that is regulated by prednisolone. E-cadherin is a predominant component of the adherens junctions that support the formation of epithelial barrier. In patient with CD, the expression of E-cadherin is downregulated in the colonic mucosa. Polymorphisms in the E-cadherin gene are also associated with CD [55]. Our data highlight the relevance of E-cadherin in the pro-inflammatory cytokine-mediated CD pathogenesis, as well as its potential contribution to the prednisolone-induced epithelial barrier augmentation. In addition, exposure of the cytokine cocktail also led to a decrease in CLDN-1 and CTNNB1, the expression of which was not affected by the incubation of prednisolone, indicating multiple mechanisms were involved upon by the cytokine and were not fully attenuated by prednisolone.

In this study, exposure of CD patient-derived intestinal organoids to the cytokine complex activated the MAPK p38 pathway, and the MLCK signaling as evidenced by increased phosphorylation of NF-kB p65 and MLC, as well as the STAT1 pathway. However, supplementation of prednisolone prevented the activation of MLCK and STAT1 signalings, but did not affect the MAPK signaling, indicating the beneficial effects of prednisolone on epithelial barrier function are mainly attributable to the activated MLCK and STAT1 pathways. Previous evidence has demonstrated a crucial role of MLCK and JAK-STAT1 pathways in the pathogenesis of CD. In CD patients, the expression of MLCK was significantly increased in intestinal tissues and correlated with the disease activity [56–58]. MLCK has also been implicated as a prominent player in the regulation of barrier junctions by different cytokines. For instance, TNF-α, IFN-γ, and IL-1β were all shown to induce MLCK activation [35, 36, 59, 60]. Increased transcriptional expression or enzymatic activity of MLCK impairs the interaction between the actin-myosin cytoskeleton and junction proteins, which subsequently disrupts the junction scaffold and eventually leads to the loss of barrier integrity. Our results further revealed that an engagement of the MLCK pathway is essential for prednisolone-induced permeability enhancement in the intestinal organoids. Furthermore, the JAK/STAT signaling pathway is highly involved in the pathological processes associated with CD. A number of pro-inflammatory cytokines contribute to CD pathogenesis through converging the JAK-STAT machinery [61]. Several small molecules that inhibit the JAK-STAT pathway have shown efficacy in early phase trails of CD treatment. In addition to its strong linkage to intestinal immunity, emerging evidence also revealed the implication of JAK-STAT signaling in intestinal epithelial barrier function. Hereby, exposure of the cytokine cocktail to the intestinal organoids led to increased phosphorylation of STAT1, which was hampered by the incubation of prednisolone, assigning a potential role of JAK-STAT1 axis in the regulation of barrier function by prednisolone.

In the current study, we did not observe a difference in the effect of cytokines or prednisolone on epithelial barrier function in intestinal organoids derived from female or male patients. However, it is worth noting that we have included only 6 inactive CD patients, being 3 male and 3 female subjects. This small size of the subgroups precluded us from performing additional analyses to investigate whether the factor of gender has an effect on the regulation of epithelial barrier function by cytokines or prednisolone. Further studies including a larger number of patients are warranted.

In conclusion, our study for the first time demonstrated that prednisolone is able to restore epithelial barrier function that was attenuated by combined cytokine TNF-α, IFN-γ, and IL-1β exposure in CD patient-derived intestinal organoid. On the molecular level, this effect is GR-dependent with a pronounced involvement of MLCK and STAT1 signaling pathway. Moreover, we revealed that supplementation of prednisolone could restore the expression of CLDN-2, E-cadherin, and ILDR-1. These findings highlight the relevance of pro-inflammatory cytokines as key pathogenic factors in inducing intestinal barrier dysfunction that contribute to disease development, identify new molecular junction targets regulated by prednisolone, and underscore intestinal barrier restoration as a potential mechanism that contributes to the clinical efficacy of prednisolone in CD patients.

## Supplementary information

ESM 1 (DOCX 697 kb)

## Data Availability

Not applicable.
