# Supplementary material for: Corticosteroid enhances epithelial barrier function in intestinal organoids derived from patients with Crohn’s disease
Source: J Mol Med (Berl). 2021 Feb 11;99(6):805–15. doi: 10.1007/s00109-021-02045-7 (PMC8164603; doi:10.1007/s00109-021-02045-7)
Supplement: Supplementary file 1 — (DOCX 697 kb) [file 109_2021_2045_MOESM1_ESM.docx]

**Supplementary Information**

**Corticosteroid enhances epithelial barrier function in intestinal organoids derived from patients with Crohn’s disease**

Pan Xu^1, 2^, Montserrat Elizalde^1, 2^, Marieke Pierik^1, 2^, Ad Masclee^1, 2^, Daisy Jonkers^1, 2*^

**Affiliation:** ^1^Division of Gastroenterology-Hepatology, Department of Internal Medicine, Maastricht University Medical Centre+, Maastricht, the Netherlands, ^2^School of Nutrition and Translational Research in Metabolism, Maastricht University, the Netherlands.

**Short title**: Corticosteroid enhances epithelial barrier in intestinal organoids

**Correspondence:** Daisy Jonkers PhD

Department of Internal Medicine, Division of Gastroenterology and Hepatology, Maastricht University Medical Centre, P. Debyelaan 25, 6229 HX Maastricht, The Netherlands.

Tel: +31-043-3884266, Fax: +31-43-3874692. Email: [d.jonkers@maastrichtuniversity.nl](mailto:d.jonkers@maastrichtuniversity.nl)


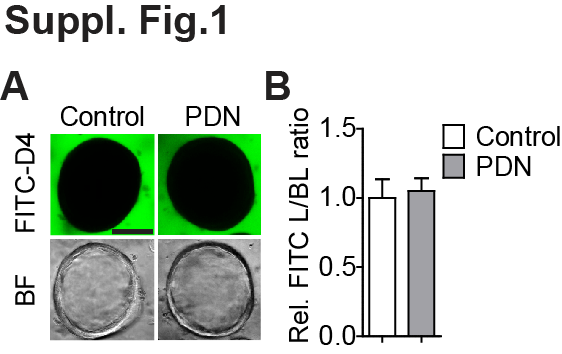


**Supplementary Figure 1.** Representative FITC-D4 permeation and bright-field (BF) microscopy images (A), and quantification of FITC-D4 permeation (B) in control and prednisolone (10 μM, 24 hours) treated intestinal organoids derived from CD patients (n = 3). The bar indicates 50 μm. The mean fluorescence intensity of FITC-D4 measured and expressed as the L/BL ratio of the luminal (L) over the basal (BL) compartment. Data expressed as means ± SEM.


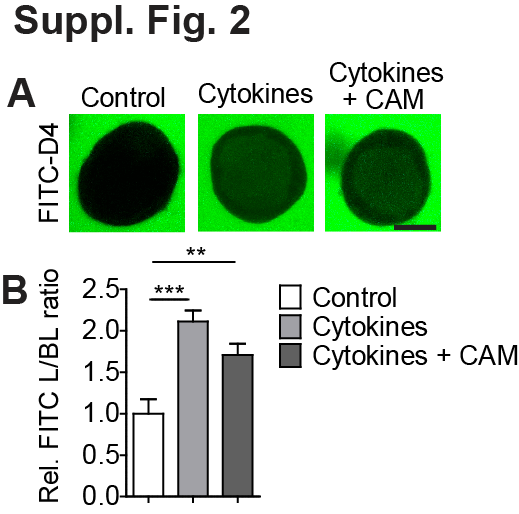


**Supplementary Figure 2.** Representative images (A) and quantification (B) of FITC-D4 permeation in CD patient-derived intestinal organoids that were exposed basolaterally with or without cytokine cocktail (20 ng/mL TNF-α, IFN-γ and IL-1β, n = 6) for 24 hours, with or without clarithromycin (CAM) incubation (80 μM, n = 6) for 12 hours. The bar indicates 50 μm. The mean fluorescence intensity of FITC-D4 measured and expressed as the L/BL ratio of the luminal (L) over the basal (BL) compartment. Data expressed as means ± SEM.


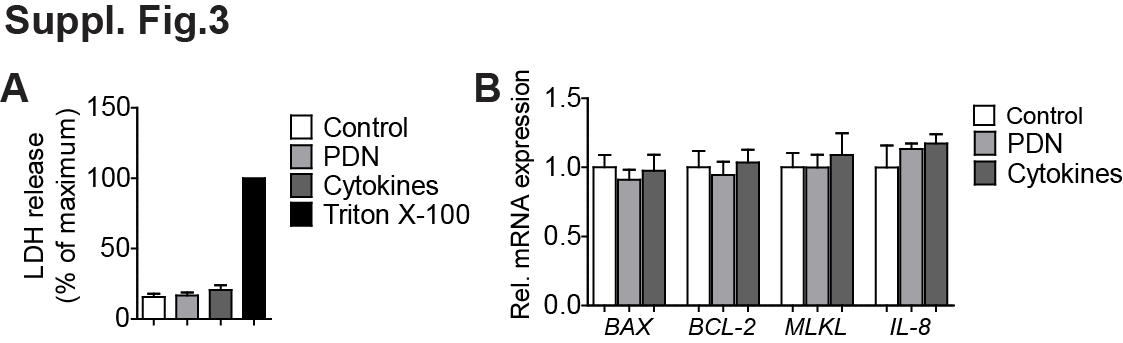


**Supplementary Figure 3.** (A) Effect of prednisolone (10 μM) or cytokine cocktail (20 ng/mL TNF-α, IFN-γ and IL-1β) treatments on LDH release in intestinal organoids derived from CD patients (n = 3). Triton X-100 was used as positive control to induce maximum LDH leakage. Data are presented as percentage of maximum LDH release. (B) Relative mRNA expression of BAX, BCL-2, MLKL and IL-8 in intestinal organoids derived from CD patients (n = 3) upon abovementioned treatments. Data expressed as means ± SEM.


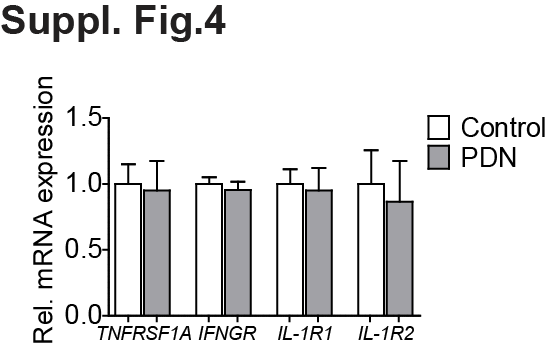


**Supplementary Figure 4.** Relative mRNA expression of TNFRSF1A, IFNGR, IL-1R1 and IL-1R2 in CD patient-derived intestinal organoids with or without prednisolone treatment (10 μM, n = 3). Data expressed as means ± SEM.


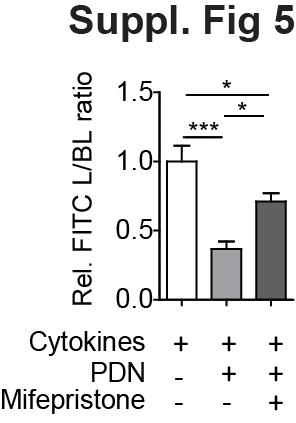


**Supplementary Figure 5.** Quantification of FITC-D4 permeation in control and cytokine cocktail (20 ng/mL TNF-α, IFN-γ and IL-1β, 24 hours) treated intestinal organoids derived from CD patients (n = 6) with or without the incubation of prednisolone (10 μM) or mifepristone (10 μM) for 12 hours. Data expressed as means ± SEM.


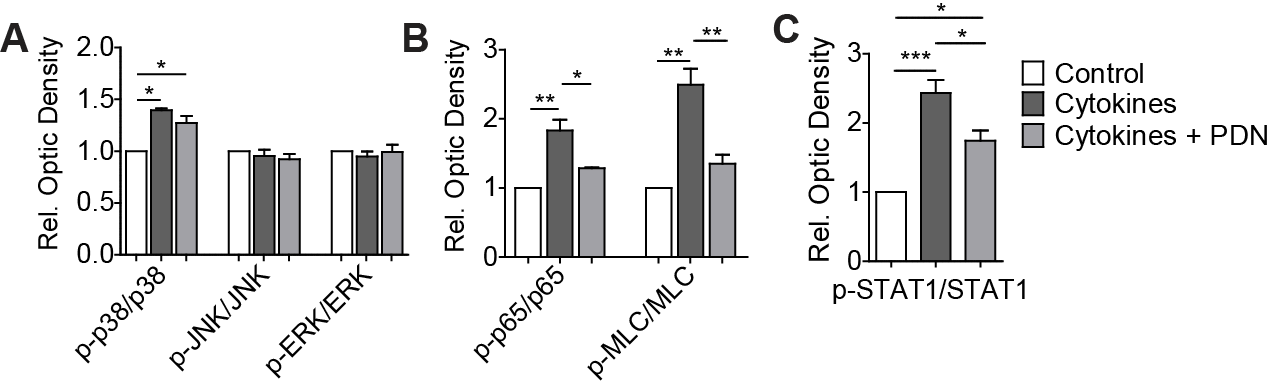
**Supplementary Figure 6.** Quantitative densitometry analysis on phosphorylation levels of p38, JNK and ERK (A), p65 (B), and STAT1 (C) in intestinal organoids derived from CD patients (n = 6) treated with or without cytokine cocktail (20 ng/mL TNF-α, IFN-γ and IL-1β, 24 hours), with or without the incubation of prednisolone (10 μM) for 12 hours. Data expressed as means ± SEM.


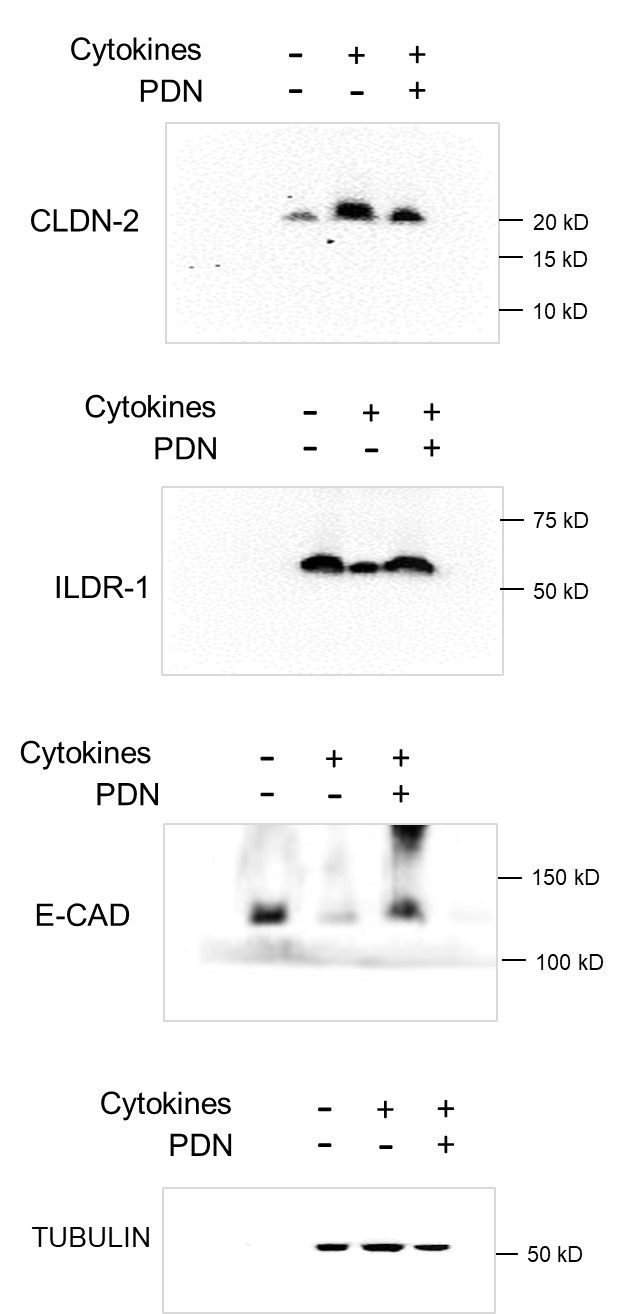


**Supplementary Figure 7.** Original Western blot images of Figure 2B.


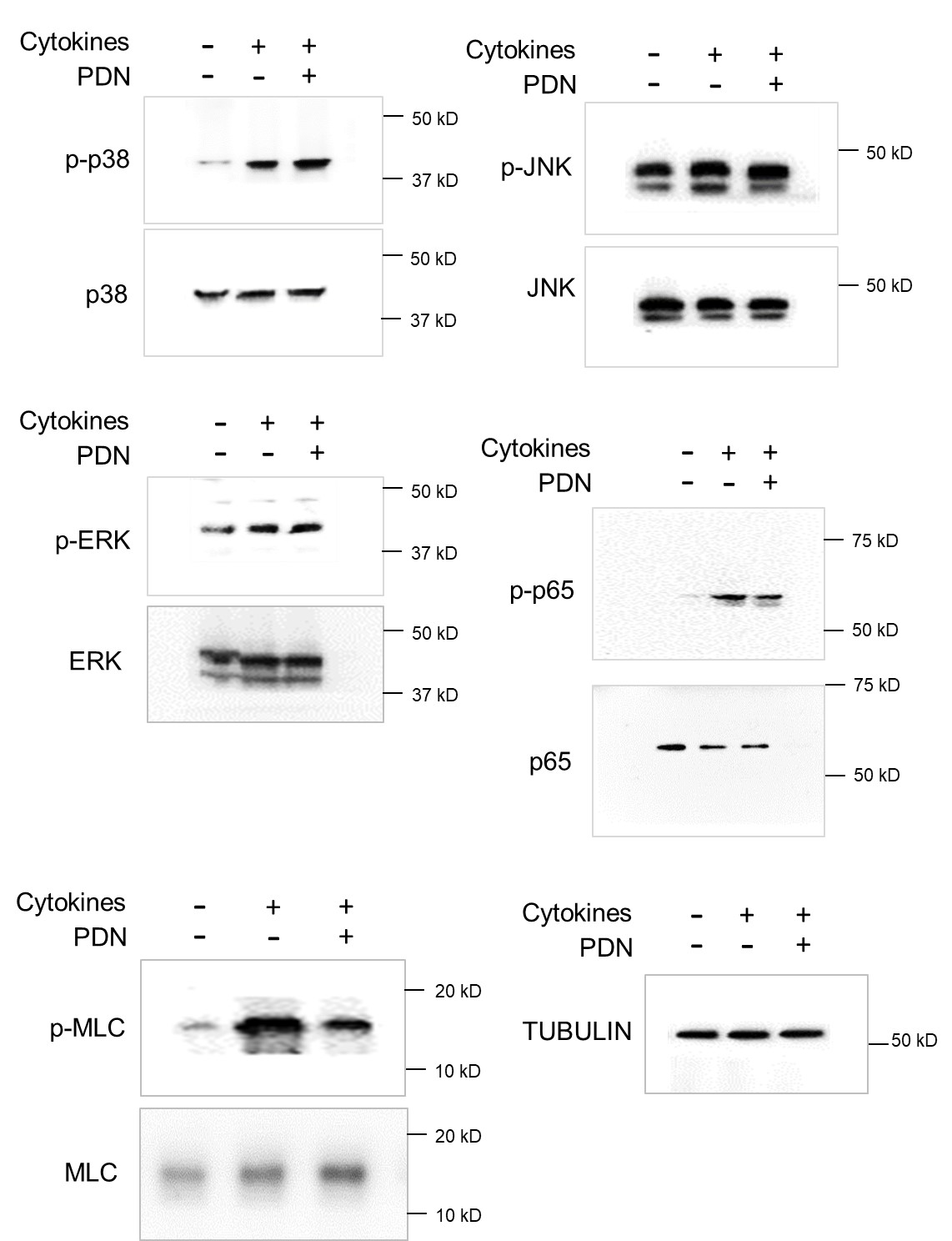


**Supplementary Figure 8.** Original Western blot images of Figure 4A and 4B.


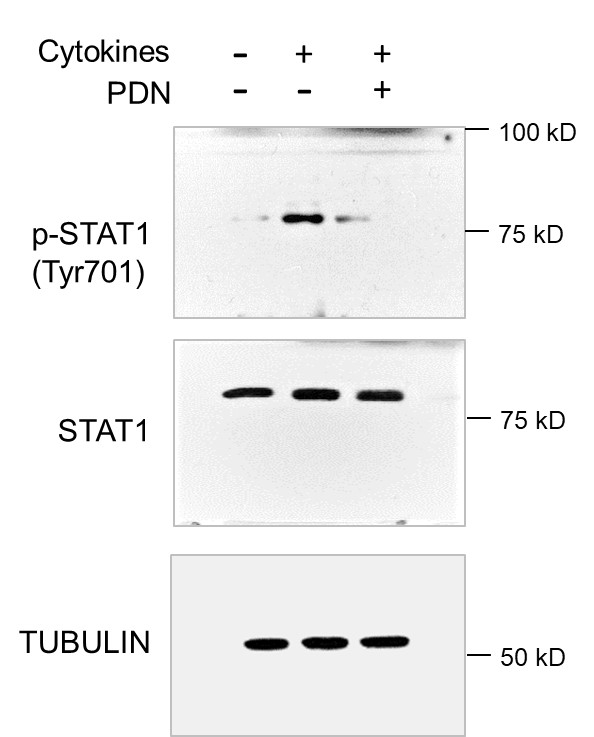


**Supplementary Figure 9.** Original Western blot images of Figure 4C.

**Supplementary Table 1**: Baseline characteristics of CD patients for biopsy collection

|  | **Inactive CD (n = 6)** |
| --- | --- |
| Age (year), mean (range) | 57,5 (51 - 68) |
| Sex (male/female) | 3/3 |
| SES-CD score | 0 |
| Colectomy | 4 |
| Age at diagnosis |  |
| A1 | 0 |
| A2 | 4 |
| A3 | 2 |
| Location of the disease^1^ |  |
| L1 | 3 |
| L2 | 1 |
| L3 | 2 |
| Disease phenotype^1^ |  |
| B1 | 3 |
| B2 | 0 |
| B3 | 3 |
| Perianal disease |  |
| P | 1 |
| Medication* |  |
| Mesalazine | 1 |
| Steroids | 1 |
| Biologicals | 4 |
| Thiopurines | 3 |

| 1 According to the Montreal classification. A1 corresponds to age at diagnosis < 16, A2 as 17-40 years, A3 to > 40 years. L1 corresponds to disease in the terminal ileum, L2 to disease in the colon, L3 to disease in the ileocolon. B1 corresponds to non-stricturing, non-penetrating, B2 to stricturing, and B3 to penetrating  * Total is higher than the number of patients as some of them receive combination of medications. |
| --- |

**Supplementary Table 2**: Sequences of the primers used for qRT-PCR analysis

| **Gene** | **Forward** | **Reverse** |
| --- | --- | --- |
| 18S RNA | GTA ACC CGT TGA ACC CCA TT | CCA TCC AAT CGG TAG TAG CG |
| TNFRSF1A | TCACCGCTTCAGAAAACCACC | GGTCCACTGTGCAAGAAGAGA |
| IFNGR | TCTTTGGGTCAGAGTTAAAGCCA | TTCCATCTCGGCATACAGCAA |
| IL-1R1 | ATGAAATTGATGTTCGTCCCTGT | ACCACGCAATAGTAATGTCCTG |
| IL-1R2 | ATGTTGCGCTTGTACGTGTTG | CCCGCTTGTAATGCCTCCC |
| ICAM-1 | GGCTGGAGCTGTTTGAGAAC | ACTGTGGGGTTCAACCTCTG |
| CLDN-1 | GGG CTG CAG CTG TTG GGC TT | GGG TTG CTT GCA ATG TGC TGC T |
| CLDN-2 | AAC TAC TAC GAT GCC TAC C | GAA CTC ACT CTT GAC TTT GG |
| CLDN-3 | TTC ATC GGC AGC AAC ATC ATC | CGC CTG AAG GTC CTG TGG |
| CLDN-4 | ACA GAC AAG CCT TAC TCC | GGA AGA ACA AAG CAG AGA G |
| CLDN-7 | GGA GAC GAC AAA GTG AAG AAG | GCC ATA CCA GGA GCA AGC |
| CLDN-12 | CTC CC CAT CTA TCT GGG TCA | GGT GGA TGG GAG TAC AAT GG |
| CLDN-15 | CCT TTG GCT TCT TCA TGG | CAG AGG TTC TCG AAG ATG G |
| TJP-1 | AGG GGC AGT GGT GGT TTT CTG TTC TTT C | GCA GAG GTC AAA GTT CAA GGC TCA AGA GG |
| CDH-1 | CAC CTG GAG AGA GGC CGC GT | AAC GGA GGC CTG ATG GGG CG |
| CTNNB1 | GTG CTA TCT GTC TGC TCT AGT A | CTT CCT GTT TAG TTG CAG CAT C |
| OCCLUDIN | TCA GGG AAT ATC CAC CTA TCA CTT CAG | CAT CAG CAG CAG CCA TGT ACT CTT CAC |
| ILDR-1 | TCCTTGCTTGTGACGGTCC | CAAAGATAGGGTCCTTGCAGAAG |
| BCL-2 | TCG CCC TGT GGA TGA CTG A | CAG AGA CAG CCA GGA GAA ATC A |
| BAX | TGG CAG CTG ACA TGT TTT CTG AC | TCA CCC AAC CAC CCT GGT CTT |
| MLKL | AGAGCTCCAGTGGCCATAAA | TACGCAGGATGTTGGGAGAT |
| IL-8 | ATGACTTCCAAGCTGGCCGTGGCT | TCTCAGCCCTCTTCAAAAACTTCTC |
| MUC2 | CTC CGC ATG AGT GTG AGT | TAG CAG CCA CAC TTG TCT G |
| MARVELD2 | TCA GAC AGA TGA TGA GCG AGA | ATG TTC CTG TCG GCT TTC C |
